# Field Study of the Comparative Efficacy of Three Pyrethroid/Neonicotinoid Mixture Products for the Control of the Common Bed Bug, *Cimex lectularius*

**DOI:** 10.3390/insects6010197

**Published:** 2015-03-18

**Authors:** Changlu Wang, Narinderpal Singh, Richard Cooper

**Affiliations:** Department of Entomology, Rutgers University, New Brunswick, NJ 08901, USA; E-Mails: nsingh@aesop.rutgers.edu (N.S.); rcooper@aesop.rutgers.edu (R.C.)

**Keywords:** *Cimex lectularius*, insecticide mixture, control

## Abstract

Three insecticide mixtures that contain two classes of insecticides (pyrethroid and neonicotinoid) were recently developed to control bed bugs. We evaluated three integrated bed bug management strategies in apartments, each using the same non-chemical control methods and one of the three insecticide mixture products: Tandem (lambda-cyhalothrin + thiamethoxam), Temprid SC (beta-cyfluthrin + imidacloprid), and Transport Mikron (bifenthrin + acetamiprid). No insecticides were applied in the Control apartments. In all apartments, we installed vinyl mattress encasements (if not already present) and applied steam to beds and other infested upholstered furniture. Insecticide sprays were applied in the three treatments. Each treatment and the Control included 8–10 occupied apartments. Re-treatment was conducted during biweekly inspections if necessary. After eight weeks, the mean (± SEM) bed bug count reduction in the Tandem, Temprid SC, Transport Mikron, and Control was 89 ± 9, 87 ± 6, 98 ± 1, and 23 ± 54%, respectively. Only Tandem and Transport Mikron treatments resulted in significantly higher population reduction than the Control at eight weeks. There were no significant differences in mean percent reduction among the three treatments (Tandem, Temprid SC, Transport Mikron) at eight weeks. Tandem spray caused significantly faster bed bug reduction than Temprid SC spray and Transport Mikron spray.

## 1. Introduction

Insecticides have always been an essential tool in urban pest management. This in part is due to people’s low tolerance for pests in homes, offices, and similar indoor environments. One pest that most people are particularly intolerant of is the bed bug, *Cimex lectularius* L. (Hemiptera: Cimicidae), which has become increasingly common during the last 10 years in the U.S. [1,2]. Bed bug bites can cause discomfort and affect mental health [3,4,5]. Once introduced, bed bug infestations may spread rapidly in multifamily housing buildings [6,7]. The sighting of a single bed bug or one insect bite can trigger the customer’s request for pest control service or do-it-yourself application of insecticides. Pest control providers almost always include insecticides as part of their bed bug management programs [8]. Do-it-yourself insecticide applications are also very common [9].

Applying insecticides to control bed bugs is often more inexpensive and convenient compared to other bed bug control methods. The formulations registered for bed bugs include spray (tank mix, ready-to-use spray, or aerosol), dust, resin strip, and fumigant, with spray formulations being most popular [10]. Among the available insecticide sprays labeled for bed bug control, pest management professionals typically use synthetic materials including pyrethroids, neonicotinoids, mixtures of a pyrethroid and a neonicotinoid, chlorfenapyr, and juvenile hormone analogs (JHAs). It is known that JHAs are ineffective at the label rate for controlling bed bugs [11] and that most field populations are resistant to pyrethroid insecticides [12]. With a limited number of insecticide classes to choose from, bed bug insecticide resistance will likely get worse in the future.

Three recently marketed insecticide mixtures that contain two classes of insecticides (pyrethroid and neonicotinoid) were developed to overcome pyrethroid resistant bed bug populations; both Temprid SC and Transport Mikron in laboratory assays and Temprid SC in a field trial showed superior efficacy against pyrethroid resistant bed bugs [13]. However, there were no data on their comparative efficacy under field conditions. The objective of this study is to comparatively assess the effectiveness of three bed bug integrated pest management programs incorporating three different insecticide mixtures in apartment buildings: Tandem (lambda-cyhalothrin + thiamethoxam), Temprid SC (beta-cyfluthrin + imidacloprid), and Transport Mikron (bifenthrin + acetamiprid). The Control apartments did not receive insecticide sprays. All apartments received steam treatment, encasements if the mattress and box springs were not already encased, and bagging of the infested bed linens and clothing.

## 2. Experimental Section

### 2.1. Study Site and Selection of Test Apartments

The field study was conducted in two apartment buildings located in an affordable housing community in Irvington, New Jersey between May and August 2013. A total of 431 apartments existed among the two buildings, 37% were one-bedroom apartments and the rest were studio apartments. *Cimex lectularius* infestations had been present in the buildings for at least three years. About 15%–25% of the units were infested at the time of the study. Most of the apartments were occupied by senior citizens of >62 years old. Prior to this study, bed bug infestations were treated by residents themselves or a housing staff using various insecticide sprays and installation of mattress encasements. Permission was obtained from the director of the housing complex prior to the study.

To select test apartments, we placed 4–12 (based on the amount of furniture and number of furniture legs) Climbup insect interceptors (Susan McKnight Inc., Memphis, TN, USA) under bed and sofa legs in each apartment with previous infestation history or current infestations. The interceptors were examined after two weeks. If the total number of bed bugs in all interceptors in an apartment were <10, a thorough visual inspection was also conducted and the total number of nymphs and adults was recorded. A total of 36 apartments (33 studio units, two one-bedroom units, one double-studio unit) with 10–300 bed bugs based on total bed bug counts in all interceptors in each apartment (21 apartments), a combination of interceptor counts and a thorough visual inspection (13 apartments), or visual inspection (2 apartments) were selected. The reason for using different methods for pre-treatment count was due to the interceptor counts were too small (<10 per apartment) in some apartments or interceptors could not be installed two weeks before treatment in some apartments. Four apartments had 100–300 bed bug counts per apartment, each was randomly assigned to one of the four treatments. The rest of the apartments had 10–77 bed bug counts per apartment, these apartments were randomly assigned to four treatments. The number of apartments in each treatment was: Tandem—9 (6 based on Climbup, 3 based on Climbup + visual), Temprid SC—10 (7 based on Climbup, 3 based on Climbup + visual), Transport Mikron—9 (5 based on Climbup, 2 based on Climbup + visual, 2 based on visual), Control—8 (3 based on Climbup, 5 based on Climbup + visual). We asked the residents whether they noticed presence of bed bugs and to stop using any insecticides.

### 2.2. Materials

The following insecticides were evaluated: Tandem (3.5% lambda-cyhalothrin, 11.6% thiamethoxam, Syngenta Crop Protection, Greensboro, NC, USA), Temprid SC (10.5% beta-cyfluthrin, 21% imidacloprid, Bayer Environmental Science, Research Triangle Park, NC, USA), and Transport Mikron (6% bifenthrin, 5% acetamiprid, FMC Corporation, Philadelphia, PA, USA). Tandem was provided by the manufacturer. Temprid SC and Transport Mikron were purchased from Univar USA (Edison, NJ, USA). Prior to the application, Tandem, Temprid SC, and Transport Mikron were diluted with water to 0.13, 0.075, and 0.11% (based on total amount of active ingredients), respectively according to the product label directions. The concentration of pyrethroid and neonicotinoid in the diluted materials were: Tandem: 0.03% and 0.10%; Temprid SC: 0.025% and 0.05%; Transport Mikron: 0.06% and 0.05%. They were applied using 1 gallon B and G low pressure sprayers (# 603469, Univar USA, Edison, NJ, USA).

### 2.3. Treatment Methods

**Figure 1:**
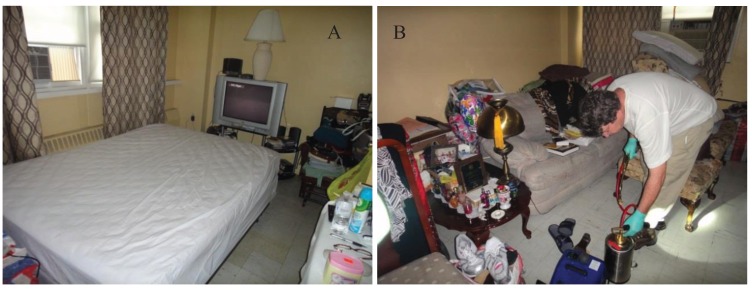
A typical test apartment. (**A**) bed area; (**B**) living area with a researcher using a B and G sprayer.

Initial treatments were conducted within 3–13 d after the initial survey. We placed the infested bed sheets, pillows, and clothing in garbage bags and asked residents to launder these items. Steam (The Steamax, Amerivap Systems, Dawsonville, GA, USA) was applied directly to bed bugs found on mattresses, box springs, bed frames, and sofas. Steam was not applied to other areas in order to evaluate the effectiveness of the insecticide sprays. In a few occasions, we used tweezers to remove live bugs found on beds or sofas when convenient. Many of the mattresses and box springs were either in the original plastic wrapping or were encased with zippered plastic encasements (Figure 1A). Zippered vinyl encasements (R.L. Plastics Inc., Avenel, NJ, USA) were installed in 14 apartments (Tandem—4, Temprid SC—1, Transport Mikron—4, Control—5) which did not have encasements or original plastic covers at the time of the initial treatment. Plastic encasements were installed during the 2 wk inspections in an additional 3 apartments (Temprid SC—1, Transport Mikron—1, Control—1).

In each apartment, except for those in the Control, insecticide spray was applied to baseboards, holes in walls, cracks and crevices around bottom of sofas, detached headboards, and other non-sleeping areas where signs of bed bugs were seen (Figure 1B). No chemicals were applied in the Control apartments. Interceptors were installed immediately after treatment following the same manner as that for the pre-treatment monitoring.

Follow-up inspections were conducted at approximately 2, 4, 6, and 8 wk. During each inspection, the numbers of bed bugs in interceptors were recorded, the bugs were dumped into toilets and flushed away, and the interceptors were re-lubricated with talc or replaced if they were damaged or dirty. Apartments with <10 bed bugs in interceptors during the initial survey were visually inspected at 2 wk for estimating bed bug counts and evaluating treatment effectiveness. Visual inspections were conducted in all apartments at 4, 6, and 8 wk. If residents were at home during inspections, the residents were asked whether they still noticed the presence of bed bugs in their homes. Additional spray and/or steam treatment was applied to live bed bugs found at 4 and 6 wk. Only steam was applied to the beds and sofas in the control apartments if bed bugs were still found by visual inspections. Therefore, the number of re-treatments in each apartment varied according to the presence or absence of live bugs detected during follow-up inspections. No re-treatment was conducted at 2 wk. At each visit, the residents were asked whether they felt bed bug bites, had laundered bed linens, and used any self-control methods. The technician time (time spent servicing each apartment multiplied by the number of technicians) and amount of pesticides used in each apartment were recorded.

### 2.4. Statistical Analysis

Bed bug counts from interceptors at each observation period were adjusted by dividing the counts by number of days since the last inspection and then multiplied by 14 to yield 14 d counts. Bed bug reduction was calculated as Reduction = 100 × (C_0_ − C_n_)/C_0_, where C_0_ is the count at 0 wk and C_n_ is the count at n wk. The changes in logarithmic transformed bed bug counts in each apartment (log (C_n_ + 1) − log(C_0_ + 1)) were analyzed using analysis of variance with repeated measurement (Proc Mixed procedure in SAS software) to determine whether there were significant differences in the bed bug count changes from 0 wk among the treatments [14]. The initial bed bug count, amount of insecticide usage, and service time were analyzed by one-way analysis of variance to determine differences among the treatments. A significance level of *α* = 0.05 is used except where indicated otherwise.

## 3. Results

The mean (± SEM) initial bed bug counts per apartment in the Tandem, Temprid SC, Transport Mikron, and Control were: 71 ± 30, 49 ± 14, 34 ± 12, and 31 ± 10, respectively. The median (min, max) bed bug counts per apartment in the four treatments were 35 (12, 100), 29 (16, 300), 16 (10, 126), and 24 (10, 118), respectively. There were no significant differences in the initial mean counts among treatments (F = 0.9; df = 3, 32; *P =* 0.43). Among the 23 interviewed residents, 30% did not notice their apartments were infested with bed bugs.

Two or three researchers serviced each apartment. The initial treatment used an average (± SEM) of 0.6 ± 0.1, 0.6 ± 0.1, and 0.5 ± 0.1 L of insecticide solution in the Tandem, Temprid SC, and Transport Mikron treatment, respectively. Mean number (± SEM) of re-treatments in the Tandem, Temprid SC, and Transport Mikron from 0–6 wk was 1.4 ± 0.2, 1.5 ± 0.2, and 1.2 ± 0.2, respectively. Average (± SEM) insecticide usage per apartment during 0–6 wk in each treatment was 0.9 ± 0.1, 1.1 ± 0.2, 0.8 ± 0.1 L, respectively. They were not significantly different (F = 1.27; df = 2, 25; *P* = 0.30). Only three apartments (one in each treatment) did not require re-treatment because bed bugs were no longer detected or ≤3 bed bugs were found during follow-up inspections. The average technician time spent per apartment (time in apartment × number of researchers) for the initial treatment in the Tandem, Temprid SC, Transport Mikron, and Control was 73 ± 8, 73 ± 9, 95 ± 14, and 68 ± 10 min, respectively. The mean technician time for servicing each apartment during 0–6 wk was 125 ± 17, 174 ± 23, 151 ± 16, and 131 ± 14 min, respectively. They were not significantly different (F = 1.6; df = 3, 28; *P* = 0.21).

**Figure 2:**
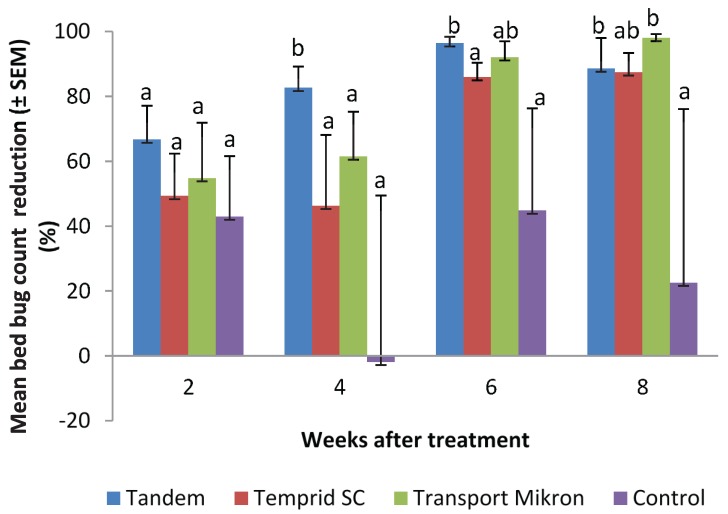
Effect of the treatments on bed bug population reduction. Bars of the same period followed by different letters indicate significant differences (*P* < 0.05).

From 0 to 8 wk, the mean (± SEM) bed bug count reduction in the Tandem, Temprid SC, Transport Mikron, and Control was 89 ± 9, 87 ± 6, 98 ± 1, and 23 ± 54%, respectively (Figure 2). At 8 wk, four apartments (Tandem—1, Temprid SC—2, Control—1) had 14–71 bed bugs per apartment. The rest of the apartments had 0–6 bed bugs per apartment. The low mean reduction and very large variance in the Control at 8 wk was due to 344% increase (from 16 to 71) in one apartment. There were significant differences among the treatments in their effect on bed bug count change (F = 5.4; df = 15, 128; *P* < 0.001), with Tandem and Transport Mikron being significantly more effective than the Control (*P* < 0.05) and no significant difference between Temprid SC and Control (*P* = 0.06) at 8 wk. If the apartment with 344% increase were excluded from the Control group, then only Tandem would be significantly different from the Control at 8 wk (F = 5.5; df = 15, 124; *P* < 0.001). In addition, Tandem caused significantly higher bed bug reduction than Temprid SC (*P* = 0.01) and Transport Mikron (*P* = 0.04) at 4 wk, and significantly higher reduction than Temprid SC at 6 wk (*P* = 0.01).

After 8 wk, 26 of the 28 apartments treated with sprays had ≤6 bed bugs per apartment. The number of apartments with zero bed bug counts based on both interceptors and visual inspections for two consecutive inspections in Tandem, Temprid SC, Transport Mikron, and Control were 2, 1, 2, and 1, respectively. Residents in these apartments did not feel bites or report seeing new bed bug activity. Therefore, bed bugs in these units were considered eliminated.

Only twenty residents from the infested apartments answered interview questions prior to the initial treatment. Thirty percent of the residents noticed bed bug bites. Among those apartments still infested with bed bugs at 8 wk, only 18% (3 out of 17) of the interviewed residents said they felt bed bug bites.

## 4. Discussion

In naturally infested occupied apartments, bed bug numbers will increase over time if left untreated [15]. Installing interceptors under furniture legs and an insecticide dust-treated band on furniture legs prevented bed bugs from increasing [15]. In this study, the bed bug counts reduced to between 0 and 5 bugs in five of the eight apartments in the Control. Therefore, steam treatment (limited to infested furniture), installing encasements and interceptors are very effective in significantly reducing bed bug populations. This result is consistent with that reported by Wang *et al*. 2012 [16]. Only one apartment in the Control experienced an increase in bed bug count after 8 wk (from 16 to 71). This increase was due to that the bed bugs on a sofa increased from 12 to 70 at 8 wk after hand removal of all visible bed bugs on the sofa at 4 wk and steam treatment at 6 wk. The sofa was not treated during 0 wk because no live bed bugs were found at that time. The resident slept on the sofa more often than on the bed after starting the treatment. The sofa had many potential bed bug hiding sites. The complex nature of the sofa furniture and the resident’s sleeping habit contributed to the failure in bed bug reduction.

The main objective of this study was to compare the three insecticide mixtures. Results show their effectiveness on bed bug population after 8 wk was comparable using the protocol defined in this study. Tandem treatment caused faster control than Temprid SC and Transport Mikron. One possible factor is that the Tandem treatment had a higher (though not statistically significant) initial count which allowed for the opportunity to kill more bed bugs. The other possible factor is that Tandem diluted spray contained twice the amount of neonicotinoid than Temprid SC and Transport Mikron. Although Transport Mikron contained twice the amount of pyrethroid than Tandem, its contribution to bed bug count reduction might be much less than the higher amount of neonicotinoid in Tandem due to the prevalence of bed bug resistance to pyrethroids. It should be noted that label-use directions for Temprid SC and Transport Mikron allow for broader application sites than Tandem, which is not allowed to be used on bed frames, mattresses, and box springs. This is one of the reasons that we incorporated steam application of the bed and sofas to allow for fair comparison of the three insecticide mixtures. At 8 wk, Tandem and Transport Mikron sprays provided significantly higher bed bug count reduction than the Control. The effect of Temprid SC spray was only significant at α = 0.1 level.

After 8 wk, elimination rates (percentages of apartments without bed bugs being detected for two consecutive visits) were low in all treatments. As in several other reported studies conducted in low income housing, there were some challenges encountered [16,17,18]. About 52% of the test apartments had significant amount of boxes, clothing, cloth bags, *etc.*, on the floors. These items may harbor bed bugs and are difficult to treat using steam or insecticide spray. Reducing the clutter could have increased the elimination rate. One resident always placed his infested pillow and clothing at various locations off the bed each day, allowing bed bugs to be scattered throughout the apartment. One apartment had a broken futon bed, which harbored many bed bugs on the mattress (which cannot be encased) and inside the bed frame tubes. Another resident re-used the dirty infested bed sheets that we placed in a plastic bag without laundering. At the beginning of this study, 30% of the interviewed residents were unaware of the presence of bed bugs in their apartments. The lack of bite symptoms is one factor related to the lack of incentive of residents to take steps in reducing clutter or laundering their linens frequently. These situations contributed to the slow reduction and failure in eliminating the bed bugs within 8 wk period. It also should be noted that the percentage of residents who were aware of bed bugs in their homes while bed bugs were still present after treatments decreased from 70% at the beginning to 18% at 8 wk. The reduction in resident awareness is apparently related to the reduction in bed bug population levels. The very high percentage (82%) of residents who failed to notice bed bugs suggests pest management professionals should not rely on resident interview to determine when to stop treatments.

Insecticide resistance may also have played a role in the low elimination rates. Bed bugs from the study site had low to medium resistance to a pyrethroid insecticide [9]. A recent study found variations among field bed bug populations in their susceptibility to pyrethroid and neonicotinoid mixtures [19]. Even after one generation, pyrethroid resistant bed bug populations exposed to Temprid SC or Transport GHP (same active ingredients as in Transport Mikron) became significantly less susceptible to Temprid SC or Transport GHP [19]. The findings suggest insecticide mixtures containing a pyrethroid and a neonicotinoid can quickly lose efficacy after repeated applications. Monitoring the development of resistance to neonicotinoids, alternate use of insecticides of different modes of action (e.g., inorganic insecticides, essential oil-based products) [9], maximize the use of non-chemical methods (such as frequent laundering, reducing clutter, applying steam, installing encasements, installing intercepting devices, etc.) [20], and conducting biweekly follow-up inspections/treatments until elimination should be planned to effectively manage bed bug infestations and to slow down insecticide resistance development.

## 5. Conclusions

A combination of steam treatment of upholstered furniture, spray application of insecticide mixtures (a pyrethroid and a neonicotinoid), and installing bed bug intercepting devices provided 87%–98% mean bed bug count reduction within 8 wk. Tandem spray provided faster control than Temprid SC and Transport Mikron spray. However there was no difference in mean bed bug count reduction among the three treatments by 8 wk. The bed bug elimination rates for all treatment groups were low after 8 wk. Future research should consider more effective incorporation of non-chemical control methods to improve results. This study was conducted in a low-income community where bed bug infestations were present for a few years and residents’ collaboration was often limited. Faster speed of bed bug elimination may be achieved in communities where resident cooperation is better.
